# Cypermethrin Induces Macrophages Death through Cell Cycle Arrest and Oxidative Stress-Mediated JNK/ERK Signaling Regulated Apoptosis

**DOI:** 10.3390/ijms17060885

**Published:** 2016-06-17

**Authors:** Fang Huang, Qiaoyun Liu, Shujun Xie, Jian Xu, Bo Huang, Yihua Wu, Dajing Xia

**Affiliations:** Department of Toxicology, Zhejiang University School of Public Health, 866 Yu-Hang-Tang Road, Hangzhou 310058, China; huangfang0794@163.com (F.H.); 21418122@zju.edu.cn (Q.L.); xieshujun@zju.edu.cn (S.X.); hyyxxj@163.com (J.X.); huangbo1991@zju.edu.cn (B.H.)

**Keywords:** cypermethrin, cell cycle arrest, apoptosis, oxidative stress, JNK/ERK

## Abstract

Cypermethrin is one of the most highly effective synthetic pyrethroid insecticides. The toxicity of cypermethrin to the reproductive and nervous systems has been well studied. However, little is known about the toxic effect of cypermethrin on immune cells such as macrophages. Here, we investigated the cytotoxicity of cypermethrin on macrophages and the underlying molecular mechanisms. We found that cypermethrin reduced cell viability and induced apoptosis in RAW 264.7 cells. Cypermethrin also increased reactive oxygen species (ROS) production and DNA damage in a dose-dependent manner. Moreover, cypermethrin-induced G1 cell cycle arrest was associated with an enhanced expression of p21, wild-type p53, and down-regulation of cyclin D1, cyclin E and CDK4. In addition, cypermethrin treatment activated MAPK signal pathways by inducing c-Jun N-terminal kinase (JNK) and extracellular regulated protein kinases 1/2 ERK1/2 phosphorylation, and increased the cleaved poly ADP-ribose polymerase (PARP). Further, pretreatment with antioxidant *N*-acetylcysteine (NAC) effectively abrogated cypermethrin-induced cell cytotoxicity, G1 cell cycle arrest, DNA damage, PARP activity, and JNK and ERK1/2 activation. The specific JNK inhibitor (SP600125) and ERK1/2 inhibitor (PD98059) effectively reversed the phosphorylation level of JNK and ERK1/2, and attenuated the apoptosis. Taken together, these data suggested that cypermethrin caused immune cell death via inducing cell cycle arrest and apoptosis regulated by ROS-mediated JNK/ERK pathway.

## 1. Introduction

Cypermethrin is the most widely used type II pyrethroid pesticide for control of agricultural and household pests [1,2,3]. Because of its stable property, low solubility in water, and long half-life, cypermethrin has become one of the most common contaminants in the environment [4]. It is known that cypermethrin mainly enters the watershed body such as runoff, which causes a significant increase in the concentration of cypermethrin in surface water [5]. Pyrethroids have been found to be extremely toxic to fish leading to tissue and organ lesions [6]. Cypermethrin usually causes damage to grass carp (*Ctenopharyngodon idellus*), such as by the necrosis of liver tissue [7]. In fish, both ion channel and ATPase have been identified as the molecular target of cypermethrin, leading to damage of normal functioning of the organs such as muscle, gills and liver in fish [8,9]. Gupta *et al.* have found severe impairment of the blood-brain barrier (BBB) development, maturation and function in mice treated with cypermethrin [10]. In addition, the reproductive toxicity of cypermethrin has also been demonstrated in a large number of animal experiments, and it has been proved that cypermethrin can cause damage to the male reproductive system, including testicular damage, sperm count, sperm motility and sperm morphology [11,12].

Excess reactive oxygen species (ROS) are produced by environmental toxicants and have been reported to induce cell death and result in human disease development. These processes include the activation of mitogen-activated protein kinase (MAPK) signaling pathways, which are observed to activate the apoptotic pathways. Previous findings revealed that cypermethrin-mediated damage of astrocytes involves Ca^2+^, ROS, c-Jun N-terminal kinase (JNK) and P38 pathways, leading to disruption of BBB and extracellular matrix molecule (ECM) development. Cypermethrin increased the intracellular ROS generation and Ca^2+^ in rat astrocytes. The JNK1/2 and P38 are subsequently activated to induce apoptosis in rat astrocyte cells [13]. Mun *et al.* also reported that cypermethrin causes oxidative stress-mediated neurotoxicity in rats, which is associated with increased ROS production [14]. Cypermethrin has also been reported to cause hepatocytes toxicity in zebrafish via oxidative stress, DNA damage and induction of apoptotic gene expression, which will facilitate to fully understand aquatic toxicological mechanism of cypermethrin in fish [15]. In African clawed frog (*Xenopus lavies*) embryos, cypermethrin resulted in DNA adduct accumulation in the presence of ultraviolet radiation b (UVB), which correlated with the induction of highly conserved genes involved in cell cycle arrest, DNA repair regulation and apoptosis (p53) [16]. The p53 protein can induce cell cycle arrest in the G0/G1 phase by upregulating the expression of p21 and promote apoptosis. Cypermethrin also produced genotoxic effects in root meristem cells of Hordeum vulgare L. according to the types and percentage of chromosomal aberrations [17].

However, the immunotoxic effects of cypermethrin on macrophages have not been well studied. To address it, we systematically examined the immunotoxicity of cypermethrin by focusing on the effects of cypermethrin on cell cycle arrest and apoptosis in macrophage-like RAW 264.7 cells. Our findings reveal a novel aspect of toxicological mechanism mediated by cypermethrin in macrophages.

## 2. Results

### 2.1. Cytotoxic Effect of Cypermethrin, Reactive Oxygen Species (ROS) Production and Apoptosis Induction in RAW 264.7 Cells

**Figure 1:**
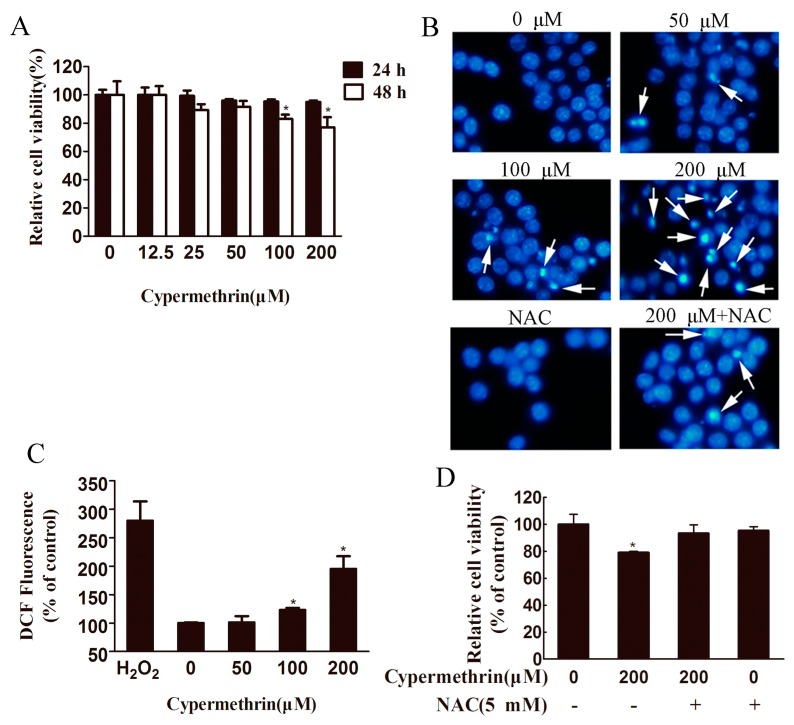
Effect of cypermethrin on cell viability and reactive oxygen species (ROS) production in RAW 264.7 cells. (**A**) The cells were treated with 0–200 μM cypermethrin for 24 or 48 h, and cell viability was assessed by 3-(4,5-dimethyl thiazol-2-yl-)-2,5-diphenyl tetrazolium bromide (MTT) assay; (**B**) Cells were plated in 6-well plates and treated with cypermethrin (0, 50, 100 and 200 μM) in the presence or absence of 5 mM NAC. 48 h later, cells were treated with Hoechst 33342 staining at 1 mg/mL for 30 min and then observed under the inverted fluorescence microscope (Original magnification, ×400); The cell nucleus change of apoptotic cell is shown by the arrows; (**C**) RAW 264.7 cells were treated with 0–200 μM cypermethrin for 1 h. Then Cells were exposed to 2,7-dichlorofluorescin diacetate (DCFH-DA) (10 μM) for 30 min. The fluorescence intensity was measured in a SYNERGY-HT multiwell plate reader at excitation and emission wavelengths of 485 and 528 nm, respectively. Untreated cells were used as negative controls and cells treated with 1 mM H_2_O_2_ as positive controls (**D**) RAW 264.7 cells were treated for 48 h with dimethylsulfoxide (DMSO), 200 μM cypermethrin only, pretreated with 5 mM *N*-acetylcysteine (NAC) before 200 μM cypermethrin treatment, or 5 mM NAC only. Then Cell viability was assessed using MTT assay. The data are presented as the means ± standard deviation of three independent experiments. * *p* < 0.05 as compared with vehicle alone.

To evaluate cypermethrin-induced cytotoxicity in macrophage cells, we first used the 3-(4,5-dimethyl thiazol-2-yl-)-2,5-diphenyl tetrazolium bromide (MTT) assay to measure the viability of RAW 264.7 cells. Treatment of RAW 264.7 cells with cypermethrin for 24 h at concentrations for up to 200 μM displayed no significant changes in cell survival compared to the control cells (Figure 1A). At 48 h, we observed significant reduction of the relative cell viability after exposure to 100 or 200 μM cypermethrin (83% ± 3% and 77% ± 7.2%, respectively). Raw 264.7 cells treated with cypermethrin at lower concentrations (from 12.5 to 50 μM) for 48 h showed no significant change in cell survival compared to the control (Figure 1A). Next, to validate the inhibitory effect of cypermethrin on cell growth was related to apoptosis, the morphologic changes of RAW cell apoptosis treated with cypermethrin (0, 50, 100 and 200 μM) were stained with Hoechst 33342. The results showed treatment with cypermethrin induced more cell apoptosis featured by nuclear fragmentation, chromatin condensation, and apoptotic body formation than the untreated control cells (Figure 1B).

In order to understand the mechanisms underlying the cytotoxic effects observed above, we focused on cypermethrin-mediated ROS generation and oxidative stress in RAW 264.7 cells. As shown in Figure 1C, the levels of intracellular ROS increased in a dose-dependent manner (50, 100, and 200 μM resulted in 101.3% ± 11%, 123% ± 3.6%, and 196% ± 22.3%, respectively, compared to control) after exposure of cells to cypermethrin for 1 h. Furthermore, pretreatment with 5 mM *N*-acetylcysteine (NAC; a potent antioxidant) partially blocked cypermethrin-induced toxicity (Figure 1D) and apoptotic body formation induced by 200 μM cypermethrin treatment (Figure 1B).

**Figure 2:**
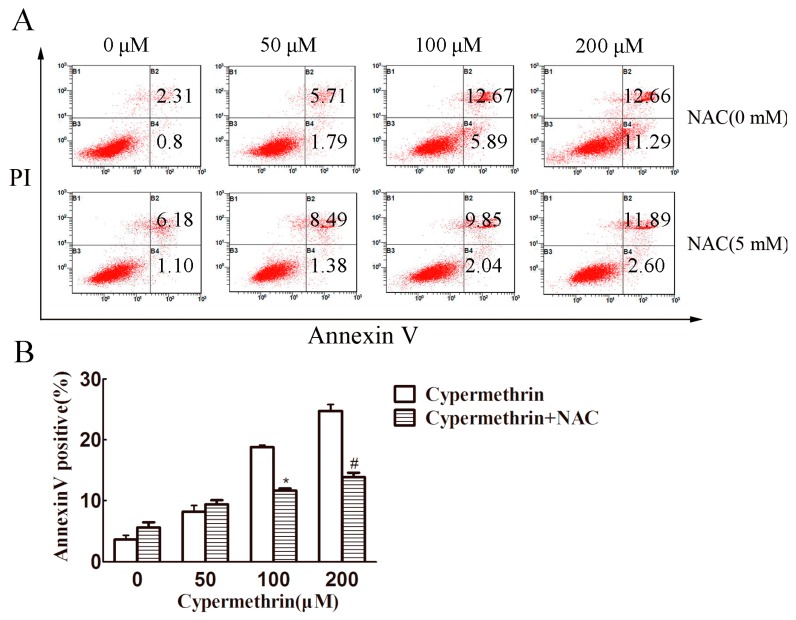
Cypermethrin induced apoptosis in RAW 264.7 cells. The cells were treated with cypermethrin in the presence or absence of 5 mM NAC for 48 h. In addition, the percentage of apoptotic cells was measured by Fluorescence Activating Cell Sorter (FACS) analysis using Annexin V/PI staining (**A**); Data were presented as means ± SD from three independent experiments (**B**). * *p* < 0.05 as compared with 100 μM cypermethrin alone. # *p* < 0.05 as compared with 200 μM cypermethrin alone.

To confirm whether apoptosis is involved in cypermethrin-induced cell death, RAW 264.7 cells were treated with cypermethrin at different concentrations for 48 h and their apoptosis was analyzed by Annexin V and propidium iodide (PI) double staining. As shown in Figure 2, higher concentrations of cypermethrin (100 and 200 μM) induce obvious apoptosis of RAW 264.7 cells, which could be partially reversed by NAC (5 mM). The results showed that the inhibitory effect of cypermethrin on the macrophage proliferation may be at least partially due to apoptosis induction, which involved cypermethrin-induced ROS production causing RAW cell death.

### 2.2. Induction of G1 Cell Cycle Arrest in Raw 264.7 Cells by Cypermethrin

**Figure 3:**
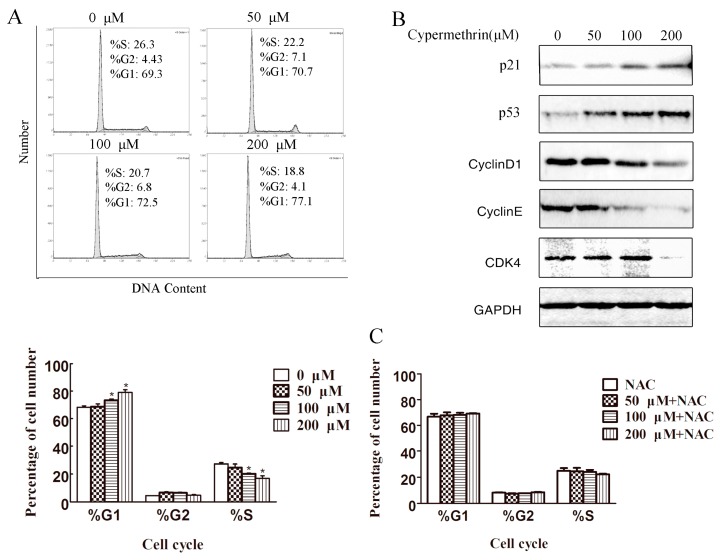
Cypermethrin leaded to G1 cell cycle arrest in RAW 264.7 cells. RAW 264.7 cells were treated with cypermethrin for 48 h (**A**). After treatment, cells were harvested and processed for cell cycle distribution analysis using flow cytometry; (**B**) Expression levels of p53 and G1 phase cell cycle regulators in RAW cells treated with cypermethrin. RAW cells were treated with or without cypermethrin for 48 h. After treatment, cells were harvested and cell lysates were subjected to the analysis of cell cycle regulatory proteins of G1-phase and p53 using western blot analysis; and (**C**) Cells were pretreated with 5 mM NAC for 1 h and treated with cypermethrin for 48 h. Cells were analyzed for cell cycle distribution. Quantitative data shown for G1, G2 and S phase cell population were representive of three independent experiments. * *p* < 0.05 as compared with vehicle alone.

After treatment with cypermethrin, cell cycle analysis was performed by flow cytometry. As shown in Figure 3A, exposure of RAW 264.7 cells to cypermethrin for 48h resulted in a significant increase in G1 phase cells in a dose dependent manner as compared to the non-treated controls (*p* < 0.05). Higher percentage of cells arrested in G1 was found when cells were treated with 200 μM cypermethrin. Treatment with 100 and 200 μM of cypermethrin for 48 h significantly up-regulated p53 protein level in RAW 264.7 cells (Figure 3B). The expression of p21 of RAW 264.7 cells treated with 100 and 200 μM of cypermethrin was up-regulated correspondingly at 48 h. As cell cycle progression is mediated by cyclin-dependent kinases (CDKs) complexed with corresponding cyclins [18], we next examined whether cypermethrin modulates the protein levels of G1 CDKs and cyclins in RAW cells. As shown in Figure 3B, cypermethrin treatment for 48 h resulted in a moderate to strong decrease in the expression of CDK4, cyclin D1 and cyclin E. Pretreatment with 5 mM NAC could partially reverse cypermethrin-induced G1 phase cell cycle arrest (Figure 3C). Together, these results suggest that cypermethrin is able to induce G1 arrest in RAW 264.7 cells.

### 2.3. Cypermethrin-Induced ROS Generation Mediated RAW Cell Apoptosis via Causing DNA Damage

**Figure 4:**
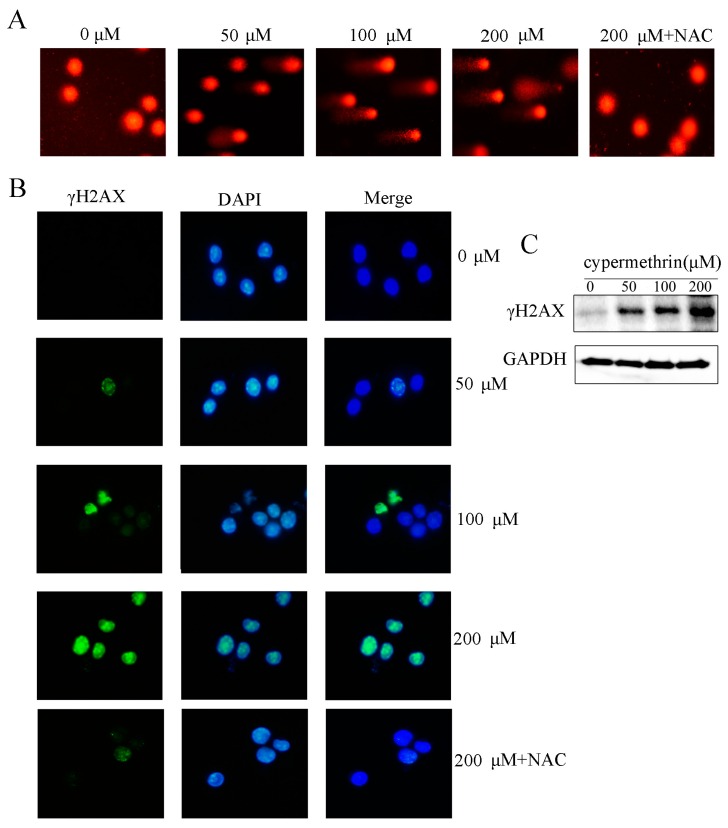
Cypermethrin-induced ROS generation triggered oxidative DNA damage. (**A**) The comet images of RAW cells when treated with 0–200 μM cypermethrin, or pretreated with 5 mM NAC before 200 μM cypermethrin treatment for 48 h; (**B**) After exposure to various concentrations of cypermethrin and pretreated with 5 mM NAC before 200 μM cypermethrin treatment for 48 h, the γH2AX-foci staining in each cell increased with higher concentrations of cypermethrin. The γH2AX foci exhibited in green stained by fluoresce isothiocyanate (FITC), and the nuclei exhibited in blue stained by DAPI. (Magnification, ×400); (**C**) The expression level of γH2AX was also examined by Western blot in RAW cells treated with cypermethrin for 48 h.

Because oxidative DNA damage is a mediator of cell death, the effect of cypermethrin-induced ROS generation on the DNA damage was investigated. After 48 h exposure to cypermethrin, we performed the Comet assay to determine whether cypermethrin induces DNA damage. Figure 4A showed that chromosomal DNA strand breaks were evident by cypermethrin at concentrations from 50 to 200 μM in RAW 264.7 cells, shown by the formation of tail DNA in cells treated with cypermethrin. Pretreating with NAC could efficiently prevent DNA damage in RAW cells by 200 μM cypermethrin treatment. Previous studies indicated that γH2AX was an early sensitive indicator of DNA double-strand breaks (DSBs) induced by chemical agents [19,20]. Here we further examined changes of γH2AX protein by immunofluorescence and immunobloting. As shown in Figure 4B, cypermethrin treatment for 48 h induced enhanced γH2AX protein levels in a dose-dependent manner. In addition, γH2AX expression by 200 μM cypermethrin exposure was attenuated in RAW cells pre-treated with NAC. Western blot analysis also showed that cypermethrin resulted in a significantly increase in the level of γH2AX as compared with the control group (Figure 4C). These findings revealed that cypermethrin-induced oxidative stress could trigger DNA damage.

### 2.4. MAPK Signaling Pathway Involved in Cypermethrin-Induced RAW Cell Apoptosis

**Figure 5:**
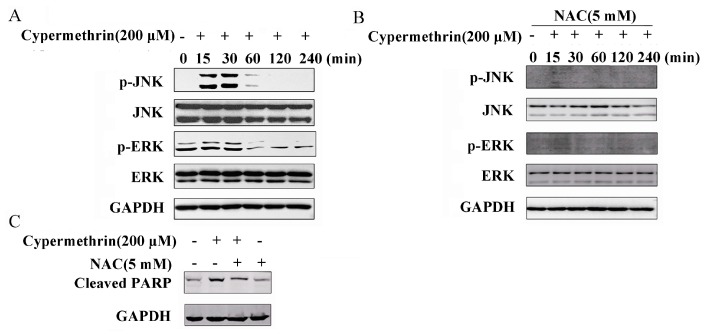
Cypermethrin induced activations of JNK and ERK MAPK signaling pathways. (**A**) RAW cells were treated with or without 200 μM cypermethrin for 15–240 min, and JNK, ERK1/2 phosphorylation levels were examined by Western blot; (**B**) Furthermore, cells were pretreated with 5 mM NAC for 1 h and exposed to cypermethrin, and the phosphorylation levels of JNK, ERK1/2 were examined by Western blot. In addition, (**C**) Cells were treated for 48 h with DMSO, 200 μM cypermethrin only, pretreated with 5 mM NAC before 200 μM cypermethrin exposure, or 5 mM NAC only. The cleaved poly ADP-ribose polymerase (PARP) was examined by Western blot.

**Figure 6:**
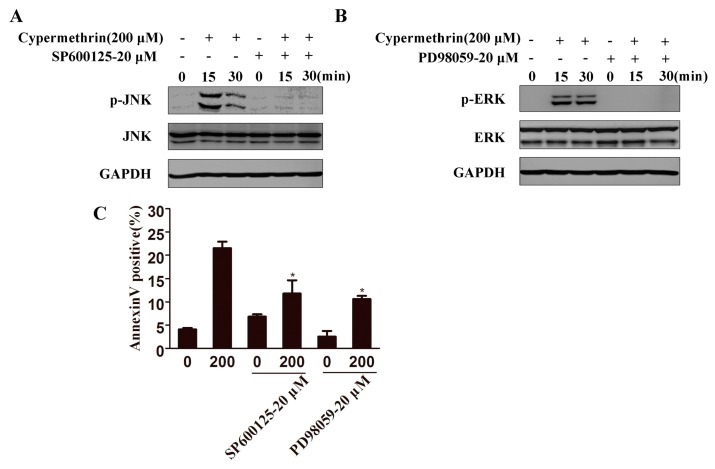
Treatment of cells with JNK/ERK-MAPK specific inhibitor prevented cypermethrin-induced apoptosis. (**A**)RAW cells were treated with JNK inhibitor (SP600125) or ERK1/2 inhibitor (PD98059) for 1 h prior exposed to cypermethrin (200 μM). The levels of JNK and ERK phosphorylation were detected by Western blot analysis (**A**,**B**); Annexin V-FITC positive cells were analyzed by flow cytometry (**C**). Data were presented as means ± SD from three independent experiments. * *p* < 0.05 as compared with treatment with 200 μM cypermethrin.

MAPK signaling pathway plays a crucial role in a variety of toxic insults-induced apoptosis, and oxidative stress is known to activate the MAPK families by protein phosphorylation [21]. Therefore, the correlation between oxidative stress and the subsequent activation of the MAPKs was investigated. As shown in Figure 5A, treatment of RAW cells with 200 μM cypermethrin for 15–30 min significantly increased the JNK phosphorylation and then rapidly decreased. ERK1/2 phosphorylation increased transiently at 15 min to 1 h and then rapidly decreased. Pretreatment with NAC could abrogate the effects of cypermethrin on the activation of JNK and ERK1/2, which indicated that ROS is critical for JNK and ERK1/2 activation induced by cypermethrin (Figure 5B). Moreover, NAC was also found to significantly reduce the PARP cleavage caused by cypermethrin (Figure 5C). The JNK inhibitor (SP600125), and ERK1/2 inhibitor (PD98059), could remarkably prevent the phosphorylation level of JNK and ERK1/2 induced by cypermethrin (Figure 6A,B). In addition, both inhibitors could effectively attenuate cypermethrin-induced cell apoptosis (Figure 6C). Therefore, these results showed that ROS production may contribute to the activation of the JNK/ERK signaling pathway and lead to RAW cell apoptosis induced by cypermethrin.

## 3. Discussion

Macrophages are highly plastic and their function dependents on the external environment. Triggering the activation of macrophages plays a major role in eliminating the invading pathogens or the alien stimulation. The classically activated macrophages produce high levels of proinflammatory cytokines such as tumor necrosis factor-α (TNF-α), interleukin-1β (IL-1β), IL-6, and reactive oxygen to protect host against the infection [22]. However, once cypermethrin causes toxicity effect in macrophage and thus affect the function of macrophage, the resolution of inflammation must be impaired. Understanding the molecular mechanism of toxical effect on macrophage may provide new insights on immunological pathology induced by cypermethrin.

The present study demonstrated that the toxicological mechanism induced by cypermethrin- in RAW 264.7 cells, involving cell cycle arrest and apoptosis. In addition, cypermethrin induced ROS generation and oxidative stress in RAW 264.7 cells. Our study confirmed that NAC pretreatment reduces the toxicity caused by cypermethrin via prevention of elevated oxidative stress. Thus, specific antioxidants may be helpful in therapy of cypermethrin-induced toxicity. DNA damage occurred in cells even at low concentration for persistent treatment. Therefore, the above will favor to understand the mechanism of cypermethrin-induced toxicity through oxidative stress, cell cycle arrest and apoptosis in macrophage.

Oxidative stress is elicited by the imbalance excessive ROS production, which can subsequently result in significant damage to cell structure. In most cases, oxidative stress is implicated in a wide variety of biological and pathological processes such as apoptosis and nervous system injuries [23]. Organisms protect themselves against the destructive effects of activated ROS by several antioxidant enzymes including catalase (CAT), glutathion peroxidase (GPx) and superoxide dismutase (SOD) [24]. NAC as an antioxidant has been reported to enhance the activity of tissue specific antioxidant enzymes such as SOD [25,26]. NAC can also inhibit the NADPH oxidase activation, a source of ROS [27]. In general, NAC is used to identify the role of ROS in various biological responses. In the current study, pretreatment with antioxidant NAC could effectively but not fully reverse cypermethrin-induced cytotoxic responses. Therefore, it demonstrated that ROS played an important role in cypermethrin-induced apoptosis.

It is generally accepted that there is a close relationship between oxidative stress and DNA damage [28]. ROS interact with DNA, potentially leading to serious consequences for the cells [29]. ROS can induce oxidative damage to DNA, including strand breaks and nucleotide modifications, especially in sequence with high guanosine content [30]. The comet assay is one of the most simple and sensitive methods for detecting DNA damage developed in recent years [31]. Phosphorylation of histone variant H2AX (γH2AX) is very important for recruitment of checkpoint proteins and DNA repair to the DNA damage sites [32,33]. Cypermethrin has been reported to induce ROS and DNA damage in adult zebrafish [15]. In our study, we evaluated the toxicity of cypermethrin using both of these methods. We found that cypermethrin -induced DNA damage in the RAW cells even at low concentrations. The comet assay revealed a dose-dependent increase of OTM, and significant γH2AX foci formation was observed after cypermethrin treatment for long time. The percentage of cells containing γH2AX foci was significantly greater compared to the control group. Western blot assay also demonstrated that the expression of γH2AX increased in a dose dependent manner. These experiments indicated that cypermethrin leaded to DNA damage in RAW cells and the damage was correlated with cypermethrin concentrations.

p53 is a tumor suppressor gene which is often dysfunctional in tumor cells [34]. Our study reported that the representative tumor suppressor p53 was up-regulated. The cyclin-dependent protein kinase inhibitor p21 is one of p53-inducible gene products [35]. It is reported that up-regulation of wild-type p53 triggers p21 protein accumulation, resulting in G0/G1 phase cell cycle arrest in MCF-7 cells [36]. The p53/p21 signal pathway is implicated in the regulation of cell cycle [37]. p53 is quickly activated to largely accumulate in the nucleus, when cells receive serious DNA damage, hypoxia and oxidative damage. Once DNA damage cannot be repaired, cells will undergo apoptosis and are then eliminated from tissues [38].

The MAPK signaling pathway is comprised of a family of protein kinases (including: JNK, ERK1/2 and p38-MAPK), which play an important role in the control of cellular differentiation, proliferation, and cell survival to death [39]. Moreover, several studies have suggested that oxidative stress can activate JNK and result in apoptosis [40,41]. However, there are few studies to investigate the toxicity of cypermethrin -induced macrophage cell death and the detailed mechanism between oxidative stress and the activation of MAPKS pathways in cypermethrin-induced macrophage cell death. The present study found that remarkably increased the expression of phosphory JNK and ERK1/2 in RAW cells, which could be reversed by NAC. Furthermore, pretreatment with NAC partially blocked the PARP activation and apoptosis. The specific JNK and ERK-MAPK inhibitor effectively attenuated cytotoxicity and apoptosis events. These results demonstrated JNK/ERK MAPK pathways were involved in the cypermethrin-induced oxidative stress triggered macrophage apoptosis.

## 4. Materials and Methods

### 4.1. Cell Culture

RAW 264.7 cells obtained from the ATCC were cultured in Dulbecco’s Modified Eagle Medium (DMEM) supplemented with 10% fetal bovine serum (Bioind, Kibbutz Beit-Haemek, Israel) and penicillin (100 U/mL)/streptomycin (100 mg/mL) under standard conditions (humidified incubator at 37 °C in 5% CO_2_/95% air). Cells were seeded to 6-, 12-, or 96-well cell culture plates and allowed to grow for 24 h prior to treatment with cypermethrin.

### 4.2. Chemicals and Antibodies

Cypermethrin, cleaved-PARP antibody and *N*-acetylcysteine (NAC) were purchased from Sigma; γH2AX antibody was purchased from Millipore (Billerica, Bedford, MA, USA); Mouse- or rabbit-monoclonal antibodies specific for phospho-JNK1/2, JNK1/2, phospho-ERK1/2, ERK1/2, p53, p21, cyclin D1, cyclin E, and CDK4 were purchased from Cell Signaling Technology (Boston, MA, USA). 2, 7-dichlorofluorescin diacetate (DCFH-DA) was purchased from Sigma (St. Louis, MO, USA). The Annexin V-fluoresce isothiocyanate (FITC)/propidium iodide (PI) apoptosis detection kit, cell cycle staining kit and glyceraldehyde-3-phosphate dehydrogenase (GAPDH) antibody were obtained from Multisciences Biotechnology (Hangzhou, China).

### 4.3. Cell Viability Assay

Cells were seeded into 96-well culture plates at a density of 1 × 10^5^ cells/well and then treated with cypermethrin in the absence or present (1 h pretreatment) of NAC (5 mM). After 48 h treatment, 20 μL (5 mg/mL) of 3-(4,5-dimethyl thiazol-2-yl-)-2,5-diphenyl tetrazolium bromide (MTT) was added to each well and incubated at 37 °C for 4 h. After the formazan was dissolved, absorbance at 490 nm was measured using an enzyme-linked immunosorbent assay (ELISA) microplate reader (TECAN Infinite M200, Bio-Rad, Hercules, CA, USA).

### 4.4. Cell Cycle Analysis

RAW 264.7 cells were seeded and treated with designed doses of cypermethrin for 48 h. After treatment, cells were trypsinized, centrifuged at 300× *g* for 5 min and washed with phosphate belanced solution (PBS), then re-suspended in 1 mL of cold 70% (*v*/*v*) ethanol, and stored at 4 °C for at least 2 h. Then, the fixed cells were washed with 2 mL PBS and incubated for 15 min. After centrifugal, the cells were added 1 mL Regent A of cell cycle staining kit. Following mixing, cells were incubated in dark condition for 30 min at 37 °C. The cells were then analyzed using a FC500 MCL machine (Beckman Coulter, Brea, CA, USA).

### 4.5. Analysis of Apoptosis

At the end of treatment with cypermethrin for 48 h in the absence or presence of 5 mM NAC, cells were harvested and washed three times with PBS, then resuspended in 500 µL binding buffer with 10 µL PI and 5 µL Annexin VFITC. Cells were incubated in the dark at room temperature for 5 min. The cells were then immediately analyzed by using a FC500 MCL machine (Beckman Coulter).

### 4.6. Comet Assay

The Comet assay has been well established for measuring DNA damage [42]. Briefly, cells were seeded in 12-well plates and treated with cypermethrin as designated. Then the cells were collected, and resuspended in 50 μL low melting point (LMP) agarose. In addition, 75 μL LMP-cell suspension was immediately pipetted onto a fully frosted microscope slide. A coverslip was then submerged in pre-chilled lysis solution (1% Triton X-100, 2.5 M NaCl, and 10 mM EDTA, pH 10) for 1.5 h at 4 °C. The slides were soaked in prechilled unwinding and electrophoresis buffer for 20 min, then were subjected to electrophoresis for 30 min at 300 mA. When electrophoresis was over, the slides were washed three times with ddH_2_O, and incubated in 70% alcohol for 5 min. Then it was stained with DAPI for 20 min, and nuclear images were visualized and captured using an Olympus AX70 fluorescent microscope (Olympus, Tokyo, Japan).

### 4.7. Immunofluorescence Microscopy

After various treatments, cells were washed with PBS and fixed in 4% paraformaldehyde for 15 min, permeabilized with 0.5% triton and blocked with 3% bovine serum albumin (BSA) for 30 min. The samples were incubated with 1:3000 mouse monoclonal anti-γH2AX antibody overnight, followed by Alexa Fluor 488-conjugated secondary antibodies for 1 h. DAPI (1 μg/mL in PBS) was added to the cells and incubated for another 30 min to stain the nuclei. The coverslip was mounted onto a glass slide, and then observed with an Olympus AX70 fluorescent microscope (Olympus).

### 4.8. Measurement of ROS Production

The level of Intracellular ROS was determined by using the 2’,7’-dichlorodihydrofluorescein diacetate (H2DCFDA) dye. Cells were seeded in a 96-well black bottom plate. After 24 h, the cells were incubated with designated concentrations of cypermethrin for 1 h. Then the medium was discarded and cells were washed with PBS. After that, cells were added with medium containing DCFDA dye (20 μM). Then cells were incubated for 30 min at 37 °C. The medium was aspirated, and 200 μL PBS was added to each well. Fluorescence intensity was measured in a plate reader at excitation of 485 nm wavelengths and emission of 528 nm wavelengths.

### 4.9. Immunoblotting

Cells were lysed with lysis buffer, then proteins were separated by 10% SDS–polyacrylamide gels (Mini-Protean II, Bio-Rad) and transferred to polyvinylidene fluoride (PVDF) Membrane (Millipore, Boston, MA, USA). After blocking with 5% non-fat milk in Tris-buffed saline with 0.1% (*v*/*v*) Tween-20 (TBST), membranes were probed with primary antibodies at 4 °C overnight, followed by incubation with horseradish peroxidase-conjugated secondary antibodies for 1 h at room temperature. After three washes, the proteins of interest were detected using an enhanced chemiluminescence kit.

### 4.10. Hoechst 33342 Staining

RAW cells were planted in 6 well plate and were treated with cypermethrin for 48 h. Cells were treated with Hoechst 33342 staining at 1 mg/mL for 30 min. Then, cells were washed twice with PBS and observed under an Olympus AX70 fluorescent microscope (Olympus).

### 4.11. Statistical Analysis

Each experiment was conducted at least three times. Data were presented as mean ± SD and statistical analysis of data was done with Student’s *t* test. *p* values < 0.05 were considered statistically significant.

## 5. Conclusions

In conclusion, this study demonstrated that cypermethrin induced cell cycle arrest and apoptosis in RAW 264.7 cells via mechanisms engaging ROS/oxidative, which caused DNA damage and subsequently JNK and ERK MAPK signaling pathways activation. Data from our study provide clear evidence showing the immunotoxic effects of cypermethrin on macrophages.
